# Distinct roles for MDA5 and TLR3 in the acute response to inhaled double-stranded RNA

**DOI:** 10.1371/journal.pone.0216056

**Published:** 2019-05-08

**Authors:** Janelle M. Veazey, Timothy J. Chapman, Timothy R. Smyth, Sara E. Hillman, Sophia I. Eliseeva, Steve N. Georas

**Affiliations:** 1 Department of Microbiology and Immunology, University of Rochester, Rochester, New York, United States of America; 2 Department of Medicine, Division of Pulmonary and Critical Care Medicine, University of Rochester, Rochester, New York, United States of America; 3 Department of Environmental Medicine, University of Rochester, Rochester, New York, United States of America; University of Kentucky, UNITED STATES

## Abstract

The airway epithelial barrier is critical for preventing pathogen invasion and translocation of inhaled particles into the lung. Epithelial cells also serve an important sentinel role after infection and release various pro-inflammatory mediators that recruit and activate immune cells. Airway epithelial barrier disruption has been implicated in a growing number of respiratory diseases including viral infections. It is thought that when a pathogen breaks the barrier and gains access to the host tissue, pro-inflammatory mediators increase, which further disrupts the barrier and initiates a vicious cycle of leak. However, it is difficult to study airway barrier integrity *in vivo*, and little is known about relationship between epithelial barrier function and airway inflammation. Current assays of pulmonary barrier integrity quantify the leak of macromolecules from the vasculature into the airspaces (or “inside/out” leak). However, it is also important to measure the ease with which inhaled particles, allergens, or pathogens can enter the subepithelial tissues (or “outside/in” leak). We challenged mice with inhaled double stranded RNA (dsRNA) and explored the relationship between inside/out and outside/in barrier function and airway inflammation. Using wild-type and gene-targeted mice, we studied the roles of the dsRNA sensors Toll Like Receptor 3 (TLR3) and Melanoma Differentiation-Associated protein 5 (MDA5). Here we report that after acute challenge with inhaled dsRNA, airway barrier dysfunction occurs in a TLR3-dependent manner, whereas leukocyte accumulation is largely MDA5-dependent. We conclude that airway barrier dysfunction and inflammation are regulated by different mechanisms at early time points after exposure to inhaled dsRNA.

## Introduction

A crucial step in the innate immune response to viral infections is the sensing of viral nucleic acid intermediates by pattern recognition receptors (PRRs) [1, 2]. Double stranded RNA (dsRNA) is a particularly potent nucleic acid intermediate and activates different PRRs including Toll Like Receptor 3 (TLR3) and members of the RIG-I like receptor (RLR) family, including Melanoma Differentiation-Associated protein 5 (MDA5) [2]. After binding to dsRNA, these PRRs induce the expression of interferons and cytokine genes, leading to tissue inflammation and anti-viral immunity [1, 3].

Although dsRNA receptors induce broadly overlapping patterns of gene expression, emerging data suggest that they have specialized roles after viral infections. For example, TLR3^-/-^ mice are particularly susceptible to herpes simplex virus infection in the central nervous system, but are surprisingly protected from lung injury and inflammation caused by influenza and other respiratory viruses ([4, 5], reviewed in [6, 7]). Mice deficient in the RLR family member MDA5 develop less airway inflammation acutely after Sendai virus infection, but this is followed by exacerbated chronic airway inflammation and hyper-reactivity later on [8]. TLR3 and MDA5 regulate dsRNA-induced airway epithelial cytokine secretion and interferon production *in vitro* [9–11], which likely explains the attenuation of inflammation after rhinovirus infection in TLR3 and MDA5 gene-targeted mice [12].

The reasons why TLR3 and MDA5 have non-redundant and potentially virus-specific roles in different organs are not well understood and are likely multifactorial. First, TLR3 and MDA5 are differentially expressed by hematopoietic cells and structural tissue cells [10, 13–16]. Second, TLR3 and MDA5 bind dsRNA in different subcellular locations: TLR3 recognizes dsRNA in endolysosomal compartments [17, 18], whereas MDA5 recognizes cytosolic dsRNA [19]. Third, TLR3 and MDA5 couple to different downstream adaptors, namely TRIF and MAVS, respectively [1]. Therefore, differences in the cell types infected and signaling cascades initiated could explain PRR-specific roles in the tissue response to respiratory viruses.

The synthetic dsRNA polyI:C has been widely used to investigate dsRNA signal transduction pathways *in vitro* and in animal models. Stromal cells are key sensors of viral infection in many tissues and respond to polyI:C stimulation [20, 21]. Airway epithelial cells express low levels of TLR3 and MDA5 at baseline, but their expression increases significantly after viral infection or polyI:C exposure [10, 13, 16, 22]. Inhalation of polyI:C in mice leads to airway inflammation and hyper-reactivity in a dose-dependent manner [23, 24]. Although TLR3 is required for polyI:C-induced airway hyper-reactivity [23], the specific roles of TLR3 and MDA5 in the airway inflammatory response to polyI:C have not been extensively studied.

In addition to causing epithelial cell cytokine and chemokine production, respiratory viruses and polyI:C also disrupt airway epithelial tight junctions, leading to epithelial barrier dysfunction [25–28]. Virus-induced epithelial barrier dysfunction is emerging as an important but understudied aspect of viral immunopathology and may promote viral replication [29]. Most research to-date in this area has been conducted using epithelial monolayers grown *in vitro*, where transepithelial electrical resistance (TEER) and permeability to macromolecules can be measured to assess the integrity of epithelial tight junctions. Less is known about how viruses or polyI:C affect the airway epithelial barrier *in vivo*, because airway barrier function is difficult to study in living animals. Furthermore, established assays of pulmonary permeability *in vivo* measure inside/out leak (e.g. transudation of serum proteins into the airspaces), and assays of outside/in leak are lacking.

We studied the acute effects of inhaled polyI:C in promoting both airway inflammation as well as epithelial barrier function in wild-type mice, compared with their TLR3^-/-^ and MDA5^-/-^ counterparts. In order to study outside/in airway barrier function *in vivo*, we adapted a protocol first described by Chen and Sun and measured trans-epithelial clearance of a macromolecule out of the airspaces and its accumulation in serum [30]. Here we report that TLR3 and MDA5 have distinct and non-redundant roles in the acute response to inhaled polyI:C, promoting barrier disruption and airway inflammation, respectively.

## Materials and methods

### Mice

Wild-type C57BL/6, and TLR3^-/-^ and MDA5^-/-^ mice on the C57BL/6 background, were obtained from the National Cancer Institute and Jackson Laboratories, respectively. Experimental and control animals were maintained independently and were age and sex matched. All animals were treated according to the Institutional Animal Care and Use Committee and Institutional Review Board approval.

### PolyI:C inhalation challenge and BAL

Wild-type and gene-targeted mice were administered 10–20 μg high molecular weight polyI:C (InvivoGen Cat#tlrl-pic; Version#11C21-MM) oropharyngeally (o.p.) for three days to induce airway inflammation. This procedure is well-tolerated and results in no overt signs of distress or weight loss. Twenty-four hours after the last challenge, mice were euthanized and the trachea was cannulated. Cells and fluid were washed out of the airspace with two installations of 750 μl phosphate buffered saline (PBS). Cell counts were analyzed by hemocytometry, and bronchoalveolar lavage fluid (BALF) were spun onto glass slides and stained with hematoxylin and eosin (FisherBrand 122–911). Cell differentials were analyzed blinded to experimental condition. Cell-free supernatants were analyzed for total protein via Bradford assay, and by ELISA for levels of albumin (Abcam), CXCL1, interleukin-6 (IL-6), CCL3 (MIP-1α) and interferon-lambda (all from R&D Systems), according to manufacturer instructions.

### Outside/in and inside/out leak assays using 4 kDa FITC-dextran

#### Outside/in leak

At 23 hrs post final challenge, and 1 hr prior to harvest, 0.2 mg 4kDa FITC-dextran (Sigma#46944) was administered o.p. to wild-type and gene-targeted mice. BALF was collected as above. Blood was collected via cardiac puncture, and the serum transferred to a new tube for analysis. Whole lung was dissociated via homogenization over a tea strainer in 1 ml PBS, and the cells were pelleted by centrifugation. The cell pellets from homogenized lungs were lysed in RIPA buffer containing protease inhibitors (Santa Cruz sc-24948). Samples were centrifuged for 12 min at 12,000 rpm at 4°C to clear debris and FITC-dextran levels were assessed using a fluorescent plate reader (Beckman Coulter DTX 880 Multimode plate reader). Tissue samples were also collected from untreated mice and processed identically to those that received FITC-dextran. These samples were then used to generate a calibration curve for each media (BALF, serum, lysate, homogenate) to convert values from fluorescence intensity to total micrograms of FITC-dextran.

#### Inside/out leak

At 23 hrs after final challenge, and 1 hr prior to harvest, we administered 4kDa FITC-dextran (Sigma#46944) by intra-peritoneal (i.p.) injection into wild-type and gene-targeted mice. BALF and serum were collected and analyzed as described above. In pilot experiments, 5 mg 4kDa FITC-dextran yielded the best signal to noise ratio, and was used in all subsequent studies.

### Statistical analysis

All values are expressed as means ± standard deviation (SD). Statistical analyses were performed using an unpaired t-test for two groups and ANOVA followed by Tukey’s test for multiple groups. A p-value of 0.05 or less was considered statistically significant. All data were analyzed using GraphPad Prism 8. In all figures * indicates p<0.05 when comparing vehicle (Veh) vs polyI:C treated mice, and # indicates p<0.05 when comparing results between different genotypes.

## Results

### MDA5, not TLR3, is required for acute neutrophil accumulation in the lung

**Fig 1:**
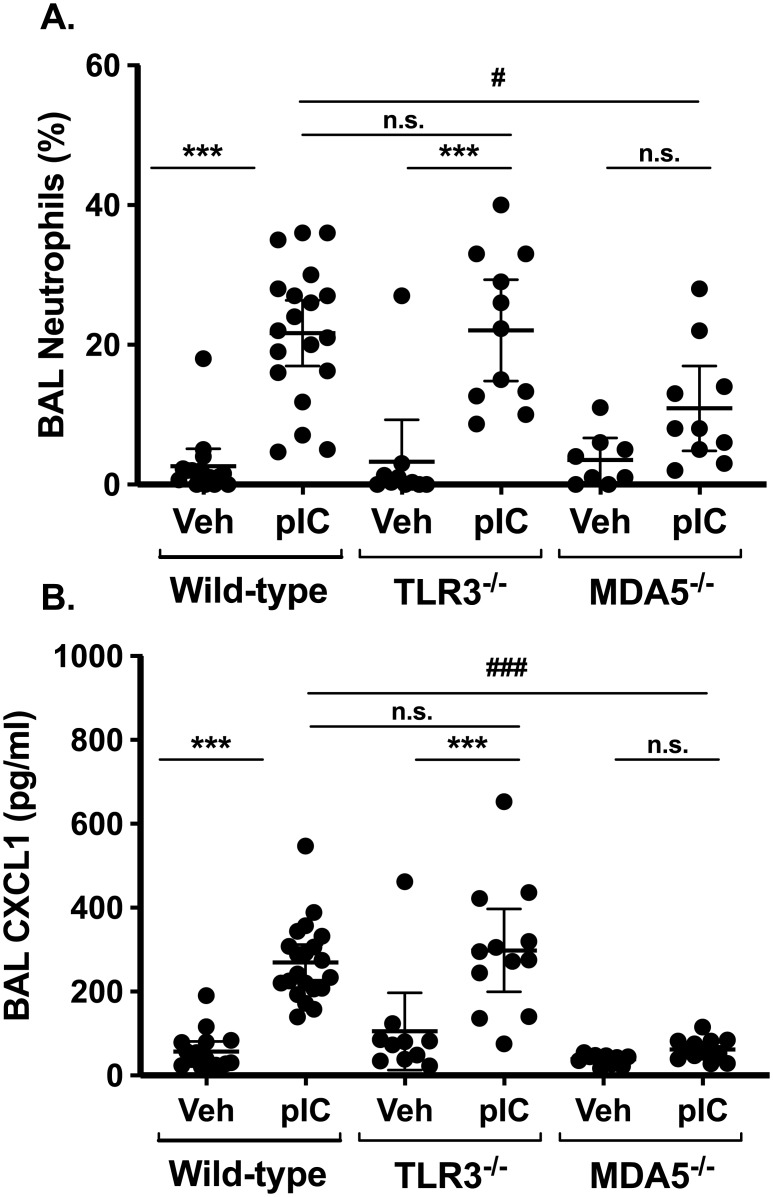
MDA5, not TLR3, promotes neutrophil accumulation and CXCL1 production after polyI:C challenge. Wild type C57BL/6 mice, TLR3^-/-^, or MDA5^-/-^ mice were administered normal saline vehicle (Veh) or 10–20 μg polyI:C (pIC) for three days. Twenty-four hours later, mice were sacrificed and bronchoalveolar lavage (BAL) was performed. BAL neutrophil percentages were determined using cytospin (A), and levels of CXCL1 were measured in cell-free supernatants by ELISA (B). Data are mean ± SD pooled from three independent experiments using both male and female mice. Statistical significance was determined by two-way ANOVA with Tukey’s multiple comparisons post-test analysis. * indicates p<0.05 when comparing Veh vs, polyI:C treated mice of a given genotype. # indicates p<0.05 when comparing wild-type vs. gene-targeted mice. ** and ## indicate p<0.01. *** and ### indicate p<0.005. n.s. = not-significant (p>0.05).

Like many respiratory viruses, polyI:C can engage either TLR3 or intracellular helicases, such as MDA5, to induce lung inflammation and anti-viral immunity. Using TLR3^-/-^ or MDA5^-/-^ mice, we assessed the role of these receptors in polyI:C-induced acute airway inflammation as monitored by neutrophil accumulation and CXCL1 levels in bronchoalveolar lavage (BAL) fluids (Fig 1). As expected, daily inhalation of polyI:C for three days resulted in neutrophil accumulation in the lungs of wild-type mice (22±10% BAL neutrophils, mean±SD) compared to mice challenged with saline alone (3±5% BAL neutrophils). In TLR3-deficient mice, neutrophils accumulated to the same degree as in their wild-type counterparts (22±11% BAL neutrophils, Fig 1A). In contrast, neutrophil accumulation was reduced by about 50% in polyI:C-challenged MDA5-deficient mice (11±8% BAL neutrophils), a significant reduction compared to wild-type (p<0.05, MDA5-deficient vs. WT). Levels of the neutrophil chemoattractant CXCL1 were markedly reduced in BAL fluids from MDA5-deficient mice compared to wild-type controls after polyI:C challenge, but CXCL1 production was not significantly affected by TLR3-deficieincy (Fig 1B).

**Fig 2:**
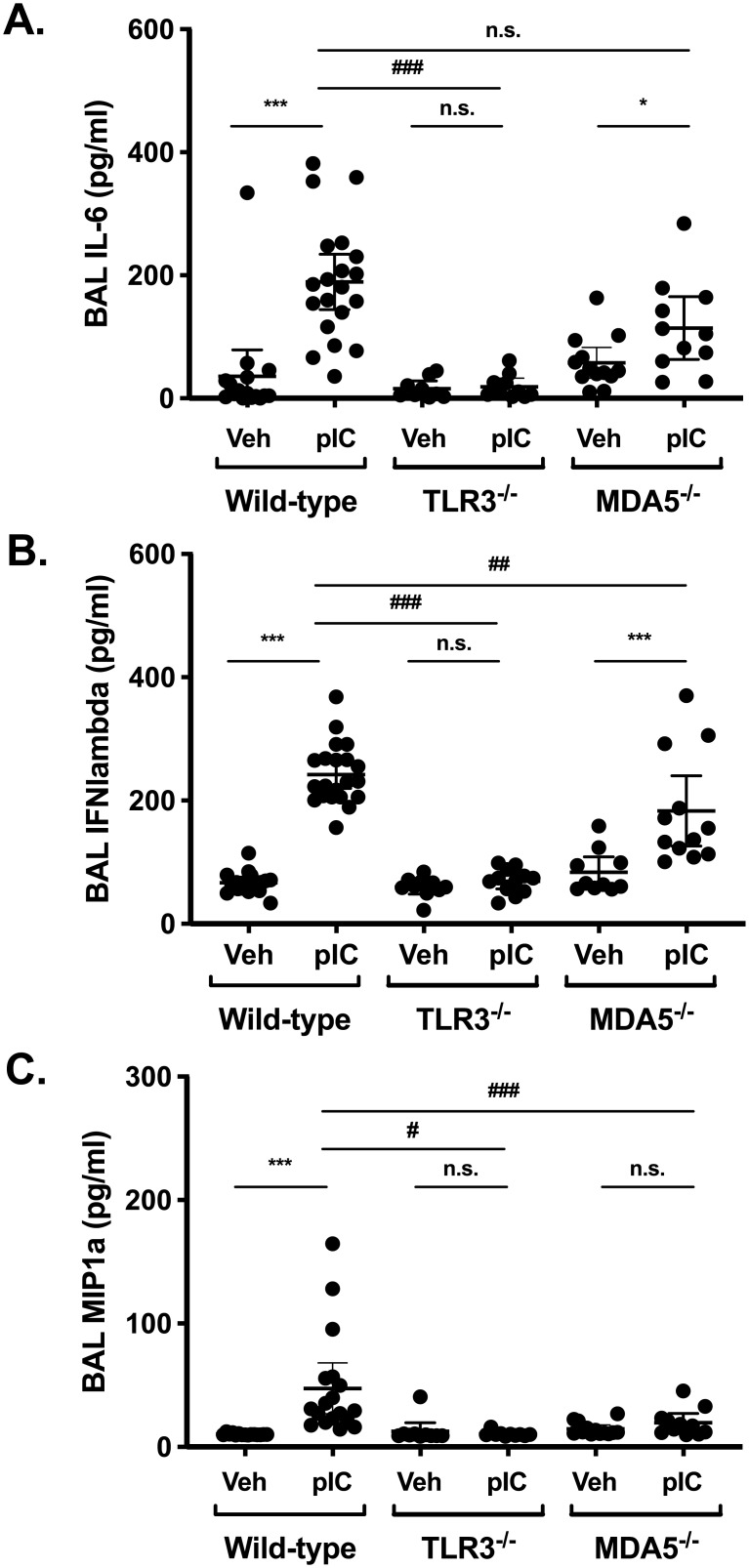
BAL levels of IL-6, INF-λ and MIP-1α are differentially affected by TLR3 and MDA5 deficiency. Wild type C57BL/6 mice, TLR3^-/-^, or MDA5^-/-^ mice were administered normal saline vehicle (Veh) or 10–20 μg polyI:C (pIC) for three days. Twenty-four hours later, mice were sacrificed and bronchoalveolar lavage (BAL) was performed. BAL levels of IL-6 (A), IFN-lambda (B), and MIP-1α (C) were measured in cell-free supernatants by ELISA. Data are mean ± SD pooled from three independent experiments using both male and female mice. Statistical significance was determined by two-way ANOVA with Tukey’s multiple comparisons post-test analysis. Statistically significant results (p<0.05) are indicated as in the legend to Fig 1.

TLR3 and MDA5 recognize dsRNA in different cellular compartments, with MDA5 recognizing cytoplasmic nucleic acids in particular. One possibility was that inhaled polyI:C might preferentially engage TLR3 in our model, such that the overall inflammatory response would be reduced in MDA5-deficieint mice. Arguing against this possibility are the observations that total BAL cell counts were similar in the different strains (not shown), and that BAL interleukin 6 (IL-6) levels were not significantly different between wild-type and MDA5-deficient mice after polyI:C challenge (Fig 2A). Interferon (IFN) lambda production was slightly reduced in MDA5-deficient mice (Fig 2B), whereas both IL-6 and IFN-lambda production were markedly attenuated by TLR3 deficiency. The chemokine MIP-1alpha (CCL3) was modestly induced after polyI:C challenge in wild-type mice, but not in TLR3- or MDA5-deficient mice (Fig 2C). We observed a significant correlation between BAL neutrophil percentages and CXCL1 levels in wild-type and TLR3-deficient mice (S1 Fig), supporting the idea that CXCL1 promotes neutrophil recruitment to the lung in this model. Taken together, these data suggest that polyI:C-induced acute airway neutrophilia is dependent on MDA5-dependent signaling pathways, whereas TLR3 is largely dispensable for this response.

### Inhaled PolyI:C disrupts pulmonary barrier integrity *in vivo* in a TLR3-dependent manner

**Fig 3:**
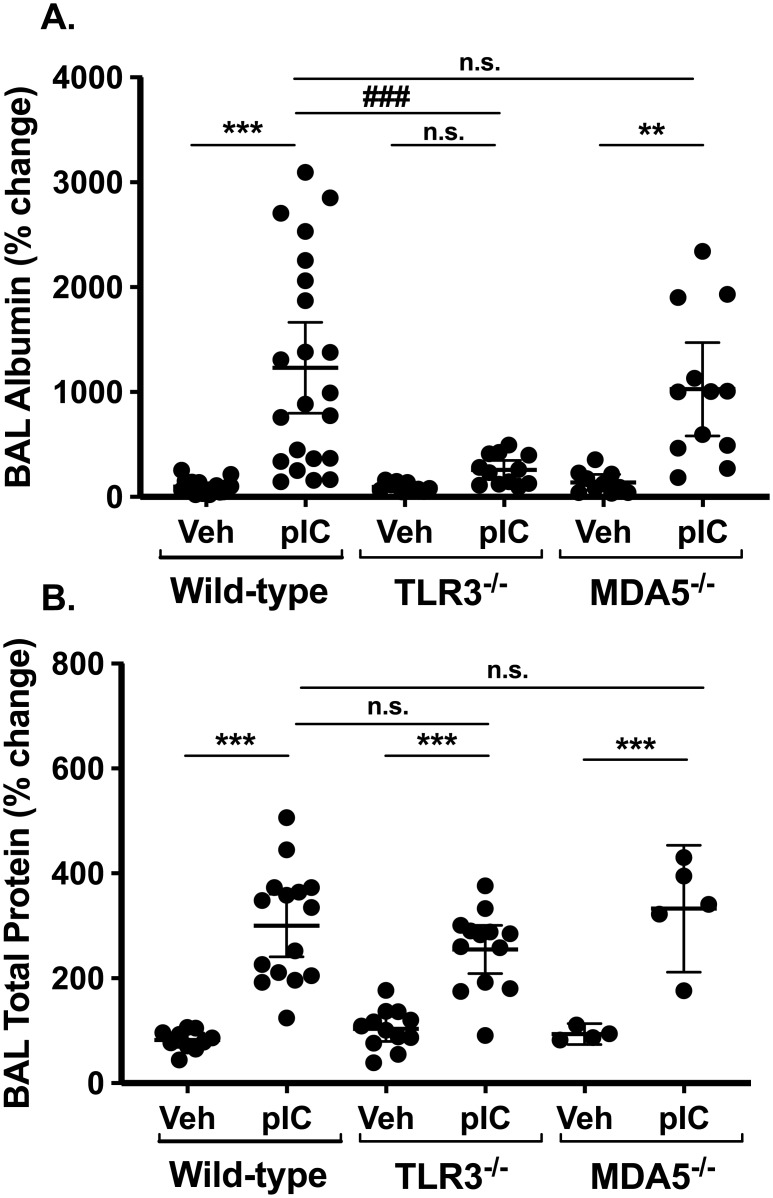
PolyI:C induces "inside/out" pulmonary leak. C57BL/6, TLR3-/- or MDA5-/- mice were challenged with saline vehicle (Veh) or 10 μg polyI:C (pIC) for three days. Twenty-four hours after the final challenge, BAL fluids were collected and analyzed for (A) albumin levels by ELISA, and (B) total protein content by Bradford assay. Statistical significance was determined by two-way ANOVA with Tukey’s multiple comparisons post-test analysis. Statistically significant results (p<0.05) are indicated as in the legend to Fig 1.

Studies of airway epithelial monolayers grown *in vitro* have revealed that both respiratory viruses and polyI:C disrupt epithelial barrier integrity by affecting the expression and function of tight junction proteins [25–28]. Epithelial barrier function has not been as well studied *in vivo*, and little is known about the role of dsRNA sensors in this process. One way to assess barrier integrity *in vivo* is to measure albumin and total protein levels in BAL fluids, which increase when the alveolar capillary membrane is injured. Fig 3 shows that BAL albumin and total protein levels increase significantly in polyI:C challenged wild-type mice. Interestingly, the increase in BAL albumin levels was significantly attenuated in TLR3-deficient mice, but not MDA5-deficient mice (Fig 3A), whereas total protein levels were not affected by either TLR3- or MDA5-deficiency (Fig 3B). Taken together with the observation that airway neutrophil accumulation is attenuated in MDA5-deficient mice (Fig 1A), these data suggest that acute airway neutrophilia and barrier integrity are regulated by distinct mechanisms after exposure to inhaled polyI:C.

### Developing a method of outside/in leak *in vivo*

**Fig 4:**
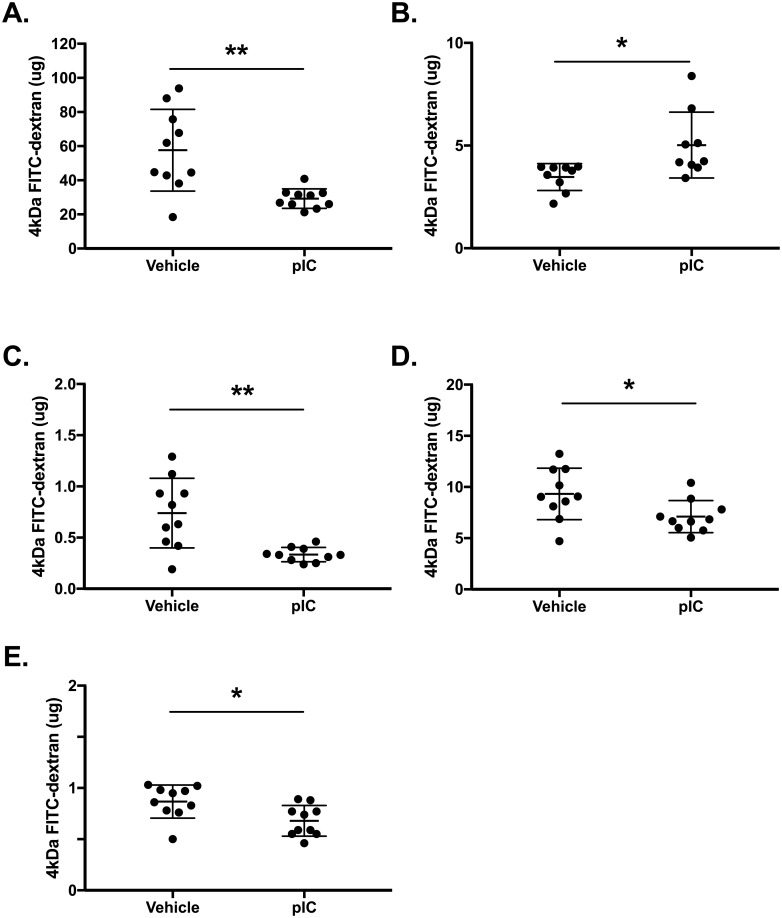
Validating a method to measure "outside-in" leak *in vivo*. C57BL/6 wild-type mice were challenged with saline vehicle or 10 μg polyI:C (pIC) for three days. Twenty-three hours after the final challenge, 0.2 mg 4kDa FITC-dextran was instilled oropharyngeally into the airway. One hour later, mice were euthanized and BAL fluids, BAL cell pellets, blood and lung tissue were collected. FITC-dextran levels were determined by fluorimetry using a standard curve to calculate total microgram amounts. Data shown are mean±SD from (A) BAL fluids, (B) serum, (C) BAL cell pellets, (D) lung tissue supernatants, and (E) lung cell pellets. BALF, serum, lung homogenate, and lysate from lung cell pellet were analyzed for. Data are mean ± SD pooled from two independent experiments (male and female). Asterisks indicate **p<0.01 and *p<0.05 using unpaired Student’s t-test.

Increased BAL albumin and protein levels after polyI:C inhalation indicates the development of “inside/out” leak, that is the transudation of a circulating protein from the vasculature into the airspaces. We also wanted to study “outside/in” leak, that is the trans-epithelial movement of inhaled molecules out of the airspaces. We reasoned that measuring outside/in leak would provide a better measurement of epithelial barrier function per se, since elevated levels of protein macromolecules in BAL fluids could occur due to endothelial or epithelial barrier dysfunction. Building on the recent study of Chen et al. in LPS-challenged mice [30], we measured the clearance of inhaled 4 kDa FITC-dextran out of the airspaces and its accumulation in serum at steady-state and after polyI:C inhalation. As expected, polyI:C-treated wild-type mice exhibited decreased FITC-dextran recovery in BALF (Fig 4A), and enhanced FITC-dextran accumulation in serum (Fig 4B), indicating the development of outside/in leak after polyI:C exposure. Since polyI:C can stimulate phagocytosis and/or endocytosis, we wanted to rule out the possibility that decreased FITC-dextran recovery in BALF was a result of cellular uptake and/or probe degradation. To investigate this possibility, we assessed FITC-dextran levels in BAL cell pellets and lung tissue homogenates. Importantly, FITC-dextran decreased in each of these compartments after polyI:C inhalation (Fig 4C–4E). Taken together with the short time between administering FITC-dextran and harvesting tissue, low probe recovery in lung cell lysates suggests that the reduced FITC-dextran recovery in the BALF is not simply due to cellular uptake and/or probe degradation.

### Outside/in leak is TLR3-dependent

**Fig 5:**
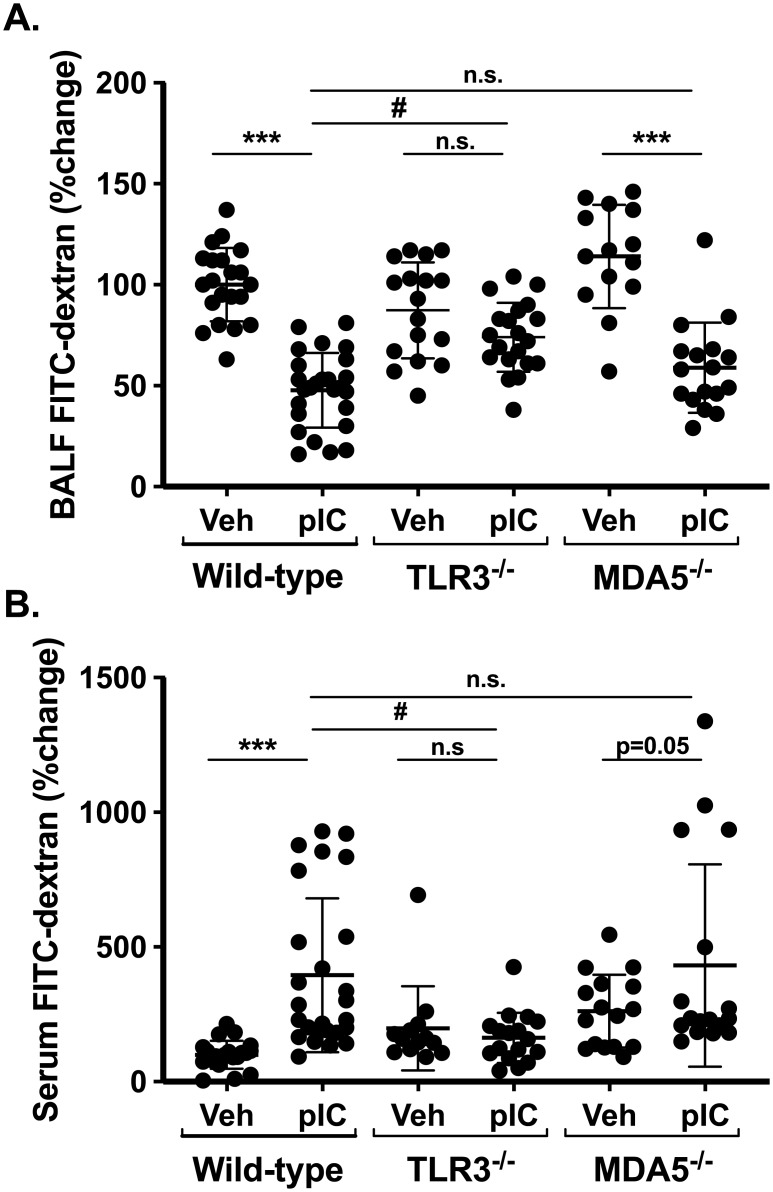
TLR3, not MDA5, regulates polyI:C-induced epithelial barrier dysfunction. Transepithelial flux of inhaled FITC-dextran was measured in wild type C57B/6 mice, TLR3-deficient, and MDA5-deficient mice using methods described in Fig 3. Data were normalized to saline challenged wild-type mice (set at 100%). PolyI:C induces rapid clearance of inhaled FITC-dextran out of the airspaces (A) and its accumulation in serum (B), indicative of reduced epithelial barrier integrity. PolyI:C-induced epithelial barrier function is attenuated in TLR3-deficient (but not MDA5-deficient) mice. Data are mean ± SD pooled from four independent experiments using male and female mice. Statistical significance was determined by two-way ANOVA with Tukey’s multiple comparisons post-test analysis. Statistically significant results (p<0.05) are indicated as in the legend to Fig 1.

Using this new method of measuring outside/in barrier function, we next measured transepithelial FITC-dextran clearance in wild-type, TLR3-deficient and MDA5-deficient mice. Fig 5A shows that after polyI:C challenge, FITC-dextran recovery in BAL fluids levels was reduced about 50% in wild-type mice, but this decrease was completely attenuated in TLR3-deficient mice. In order to control for experimental variability in BAL FITC-dextran recovery, data in Fig 5 are expressed relative to wild-type mice challenged with saline alone (set at 100%). Similarly, whereas serum FITC-dextran levels increased in polyI:C-challenged wild-type mice, this did not occur in TLR3-deficient mice (Fig 5B). We noted that steady-state leak of FITC-dextran out of airspaces was slightly higher in TLR3 deficient mice compared to wild-type (BAL recovery of 83 ± 20 vs. 59 ±13 μg in wild-type vs. TLR3^-/-^ vehicle-treated mice, p<0.01, Fig 5A). In contrast to TLR3-deficeint mice, results from polyI:C-challenged MDA5-deficient mice were similar to their wild-type counterparts both in terms of FITC-dextran clearance out of the lung (Fig 5A) and its accumulation in serum, although serum values of FITC-dextran were more variable in MDA5-deficient mice (Fig 5B).

**Fig 6:**
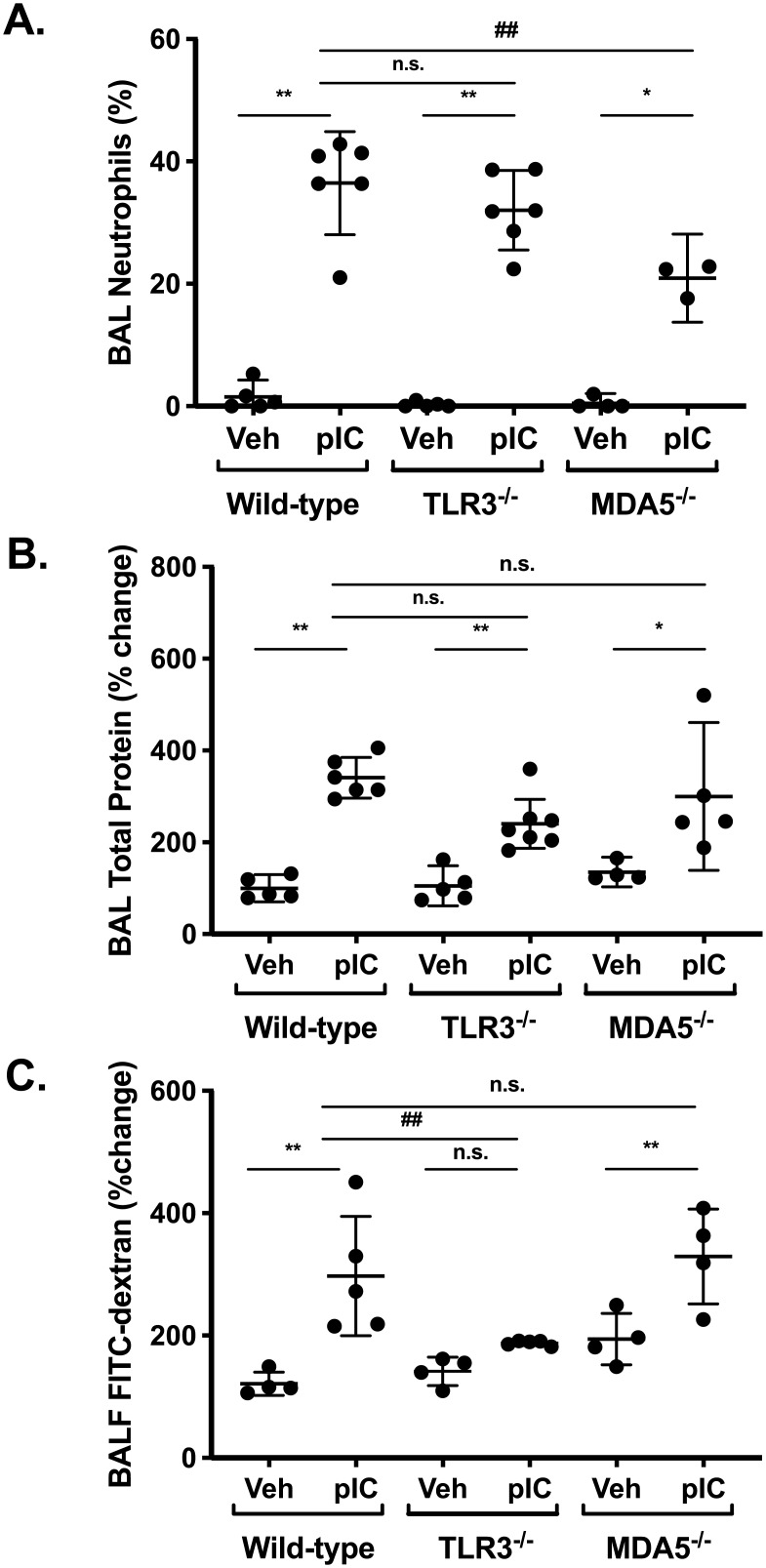
Inside/out barrier function assayed measured after FITC-dextran i.p. injection is also TLR3-dependent. Wild-type and gene-targeted mice were challenged with vehicle (Veh) or inhaled polyI:C (pIC) for three days. FITC-dextran (4 kDa, 5 mg) was injected intra-peritoneally (i.p.), and 1 hour later mice were sacrificed and BAL fluids were collected and analyzed for neutrophil percentages (A), total protein content (B), or relative fluorescence (C, normalized to saline challenged wild-type mice). Data are mean ± SD pooled from one experiment, representative of two. Statistical significance was determined by two-way ANOVA with Tukey’s multiple comparisons post-test analysis. Statistically significant results (p<0.05) are indicated as in the legend to Fig 1.

Measuring albumin and protein accumulation in lung BAL fluids is a well-established method to assess barrier integrity, but serum proteins can accumulate in the lung when either endothelial or epithelial barrier function is compromised. The rapid clearance of FITC-dextran out of the airspaces is a more direct measure of epithelial barrier function per se, but this does not allow direct comparison to measuring BAL levels of albumin or total protein that accumulate over hours / days. Furthermore, serum albumin is significantly larger than the FITC-dextran probe used in our experiments (60 vs. 4 kDa). In order to directly compare inside/out and outside/in leak using 4kDa FITC-dextran, we challenged a separate group of wild-type and gene-targeted mice with inhaled polyI:C for three days, and then administered 4 kDa FITC-dextran by intra-peritoneal (i.p.) injection and measured its accumulation in blood and BAL fluids one hour later (similar timing to Fig 5). Similar to earlier results, we found that: (i) BAL neutrophils were reduced by ~50% in MDA5-deficient mice, compared to their wild-type or TLR3-deficient counterparts (Fig 6A), and (ii) BAL total protein levels were similarly increased after polyI:C challenge in all groups (Fig 6B). Using this protocol, serum FITC-dextran was detectable in all groups after challenge with either vehicle or polyI:C, and for reasons that are currently not clear, higher serum values were observed in poly:C-challenged MDA5-deficient mice (S2 Fig). Interestingly, significantly more FITC-dextran accumulated in BAL fluids after poly:C challenge in wild-type and MDA5-deficient mice, but not TLR3-deficient mice (Fig 6C). Take together with previous results, these data indicate that acute inhalation challenge with polyI:C disrupts airway barrier integrity in a TLR3-dependent manner.

## Discussion

Activation of innate immunity by viral dsRNA involves multiple pattern recognition receptors, including TLR3 and MDA5. Emerging data suggest that these dsRNA sensors have compartmentalized roles in the development of tissue inflammation and anti-viral immunity, but it is currently unclear if they regulate distinct aspects of lung mucosal inflammation. We studied the acute response to inhalation challenge with polyI:C in TLR3^-/-^ and MDA5^-/-^ mice, compared with their wild-type counterparts. We measured airway inflammation (BAL neutrophils and CXCL1 levels) as well as barrier function, using assays of both inside/out as well as outside/in barrier integrity. We found that TLR3^-/-^ and MDA5^-/-^ have distinct roles in the acute response to inhaled polyI:C, promoting epithelial barrier disruption and neutrophilic airway inflammation, respectively. Here we consider the potential mechanisms for these observations and their implications for our understanding of respiratory virus-induced lung injury.

Our finding that acute airway neutrophilia was not affected by TLR3-deficiency in polyI:C-challenged mice are similar to the results of Stowell et al. [23]. These authors reported no significant differences in lung neutrophil recruitment in wild-type vs. TLR3^-/-^ mice after three daily challenges with 100 μg polyI:C. Because BAL levels of CXCL1 were equally induced by polyI:C in both wild-type and TLR3^-/-^ mice, we speculate that the acute production of neutrophil chemoattractants is not TLR3-dependent in our model. One implication of our findings is that previous studies attributing polyI:C-induced CXCL1 production to TLR3 engagement need to be re-evaluated [31, 32]. Additionally, Stowell et. al. [23] reported TLR3-deficiency protected mice from polyI:C-induced changes in lung function, akin to our current finding that TLR3 mediates changes in outside/in barrier function of the lung.

In contrast we found that polyI:C-dependent CXCL1 production and lung neutrophilia were dependent on MDA5, since they were both significantly attenuated in polyI:C challenged MDA5^-/-^ mice compared to wild-type controls. MDA5 is a member of the RLR family of intracellular helicases and recognizes long dsRNA within the cytoplasm. Our findings are similar to the phenotype reported by Kim et al. in MDA5^-/-^ mice infected with Sendai virus, where lung neutrophil numbers and CXCL1 production were significantly reduced at early time points after infection [8]. One potential explanation for the preferential requirement for MDA5 (and not TLR3) in airway neutrophilia after acute polyI:C exposure is that only MDA5 was engaged in our acute inhalation model. However, we do not favor this explanation for two reasons. First, it seems likely that inhaled polyI:C will be targeted to TLR3-containing endolysosomal compartments in at least some cells within the respiratory tract. Second, we found that both IL-6 production and airway barrier function were dependent on TLR3. Therefore, it seems more likely that MDA5 preferentially induces the production of neutrophil attracting chemokines, including CXCL1, during the acute response to dsRNA in the airway.

Although it was not required for polyI:C-induced airway inflammation, TLR3 was essential for polyI:C-induced epithelial barrier dysfunction, which we assessed by measuring FITC-dextran clearance out of the air spaces as well as accumulation of albumin in BAL fluids. Similar to recent studies of lipopolysaccharide challenged or respiratory syncytial virus infected mice [30, 33], we found that polyI:C challenge also induced rapid movement of FITC-dextran out of the airway, and that this was entirely dependent on TLR3. In contrast, MDA5-deficiency had no effect on this or other assays of barrier integrity. Interestingly, BAL protein increased after polyI:C challenge in both TLR3-deficient as well as MDA5-deficient mice. This result underscores the importance of using multiple assays of lung barrier function in vivo, and suggests that measuring FITC-dextran clearance will provide new insights into epithelial barrier regulation in vivo.

Although there are well-established methods to measure airway epithelial barrier integrity of cell culture monolayers *in vitro*, there is no consensus about the best way to study barrier function of the lung *in vivo*, and different assays offer different insights. Measuring protein macromolecule accumulation in BAL fluids is a widely used approach, but this is a measure of inside/out flux of molecules from the vasculature, across both endothelial and epithelial barriers, into the airway lumen. When considering how inhaled pathogens and particles gain access to lung tissues, a direct assay of outside/in transepithelial permeability is desirable, which is exactly what FITC-dextran clearance measures. While we were concerned that loss of FITC-dextran from BALF could simply be due to phagocytosis or degradation of the probe by lung cells, three observations suggest that this was not the case. First, the amount of FITC-dextran present in lung cell homogenates was reduced (and not increased) after polyI:C challenge. Second, FITC-dextran concentrations increased in serum after polyI:C exposure. Third, the total amount of probe measured in different compartments was not compatible with large scale probe degradation in the one hour time frame between FITC-dextran inhalation and obtaining BAL samples. Therefore, quantifying the recovery of inhaled FITC-dextran in BALF appears to be a valid measure of airway epithelial barrier integrity per se in living animals.

We noticed that FITC-dextran efflux from the airways of saline-challenged TLR3^-/-^ mice was slightly greater than in wild-type controls (Fig 5A). This was not associated with reduced baseline levels of BAL albumin or total protein levels, and suggests that TLR3 signaling might be required for the development of full airway epithelial barrier function at steady-state. Precedence for the idea that TLR3 has a barrier-enhancing role comes from a recent study of mouse epidermal barrier function [34], and is consistent with evidence that TLR3 transduces restorative or repair signals in some contexts [35].

It is tempting to speculate that polyI:C causes epithelial barrier dysfunction in a TLR3-dependent manner by disrupting epithelial tight junction assembly. This would be consistent with our previously published *in vitro* work demonstrating polyI:C promoted protein kinase D-dependent disassembly of tight junctions [26]. We also found that FITC-dextran accumulation in BAL after i.p. injection was markedly attenuated by TLR3-deficiency. Therefore, using multiple different approaches to measure both “inside/out” and “outside/in” barrier function, these data strongly support the conclusion that airway barrier function is acutely regulated by TLR3-dependent signaling mechanisms. It is interesting to note that preservation of airway barrier function in polyI:C challenged TLR3^-/-^ mice occurred despite the presence of robust airway neutrophilia. This suggests that barrier dysfunction in our model is not simply due to neutrophil-dependent epithelial lung injury, but rather a distinctly regulated process. Future studies using new imaging techniques to investigate the precise anatomic location of epithelial barrier dysfunction *in vivo* should help decipher the specific molecular mechanisms involved.

Out study has some limitations. First, we restricted our analysis to an early time point after polyI:C challenge, and it remains possible that we would have obtained different results at later stages of the inflammatory response to dsRNA, or during its resolution. Second, it remains to be seen whether the results we obtained using polyI:C will extrapolate to mice infected with a live replicating virus. Third, we did not identify with certainty the precise molecular mechanisms by which polyI:C leads to dysfunction of epithelial junctional complexes. Future studies will be needed to investigate these mechanisms in more detail.

In conclusion, we found that TLR3 is a key regulator of epithelial barrier integrity in the lung, but it is not required for polyI:C-induced acute airway inflammation. In contrast, MDA5 has an essential and non-redundant role in promoting airway neutrophilia, but it is not involved in acute barrier regulation. Our results highlight the utility of using multiple assays of barrier integrity *in vivo* and underscore the notion that epithelial barrier function is tightly regulated via specific signaling pathways.

## Supporting information

S1 Fig Correlation analyses between BAL neutrophil percentages and BAL CXCL1 levels in wild-type, TLR3-, and MDA5-deficient mice. (PDF)

S2 Fig Serum levels of FITC-dextran in wild-type, TLR3-deficient, and MDA5-deficient mice. (PDF)
