# Supplementary material for: Distinct roles for MDA5 and TLR3 in the acute response to inhaled double-stranded RNA
Source: PLoS One. 2019 May 8;14(5):e0216056. doi: 10.1371/journal.pone.0216056 (PMC6505938; doi:10.1371/journal.pone.0216056)

# Fig. S1

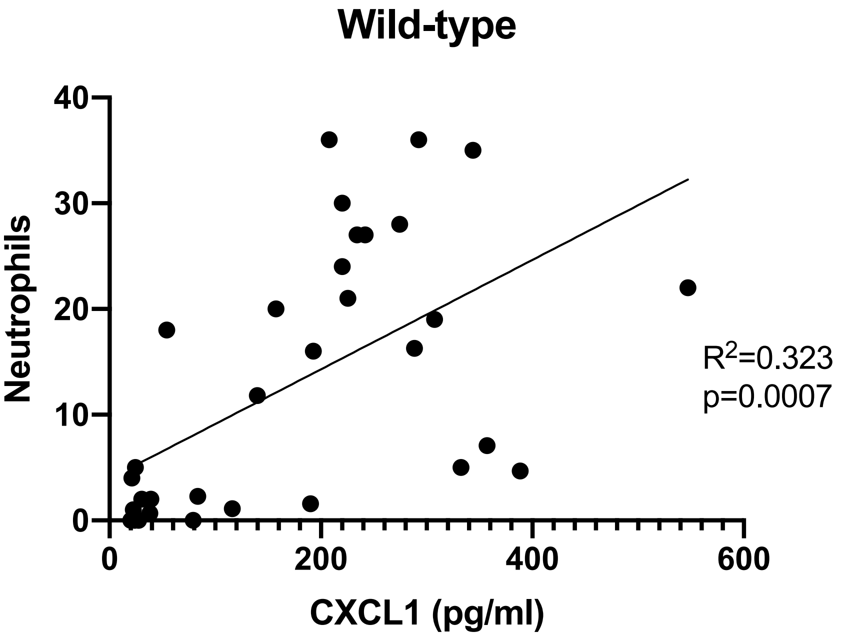


**Figure S1 Legend.** Correlation analyses between BAL neutrophil percentages and BAL CXCL1 levels in wild-type, TLR3-, and MDA5-deficient mice.

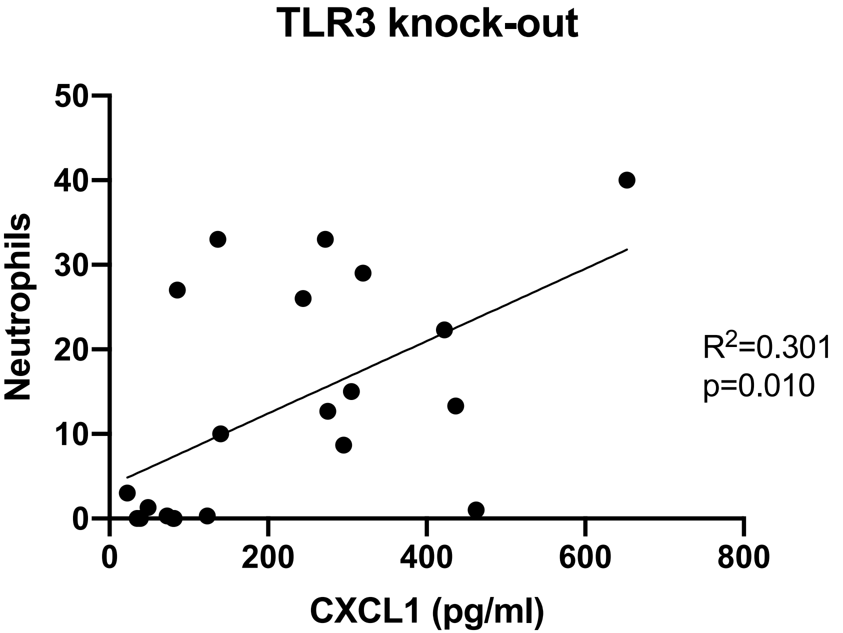


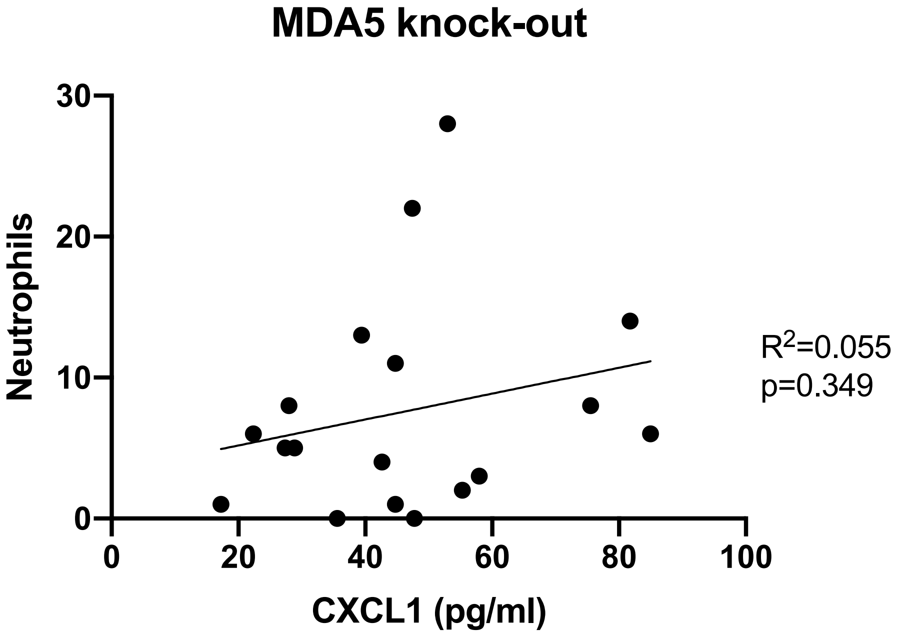

Supplement: S1 Fig — (PDF) [file pone.0216056.s001.pdf]
