# Supplementary material for: Distinct roles for MDA5 and TLR3 in the acute response to inhaled double-stranded RNA
Source: PLoS One. 2019 May 8;14(5):e0216056. doi: 10.1371/journal.pone.0216056 (PMC6505938; doi:10.1371/journal.pone.0216056)
Supplement: S2 Fig — (PDF) [file pone.0216056.s002.pdf]

Fig. S2

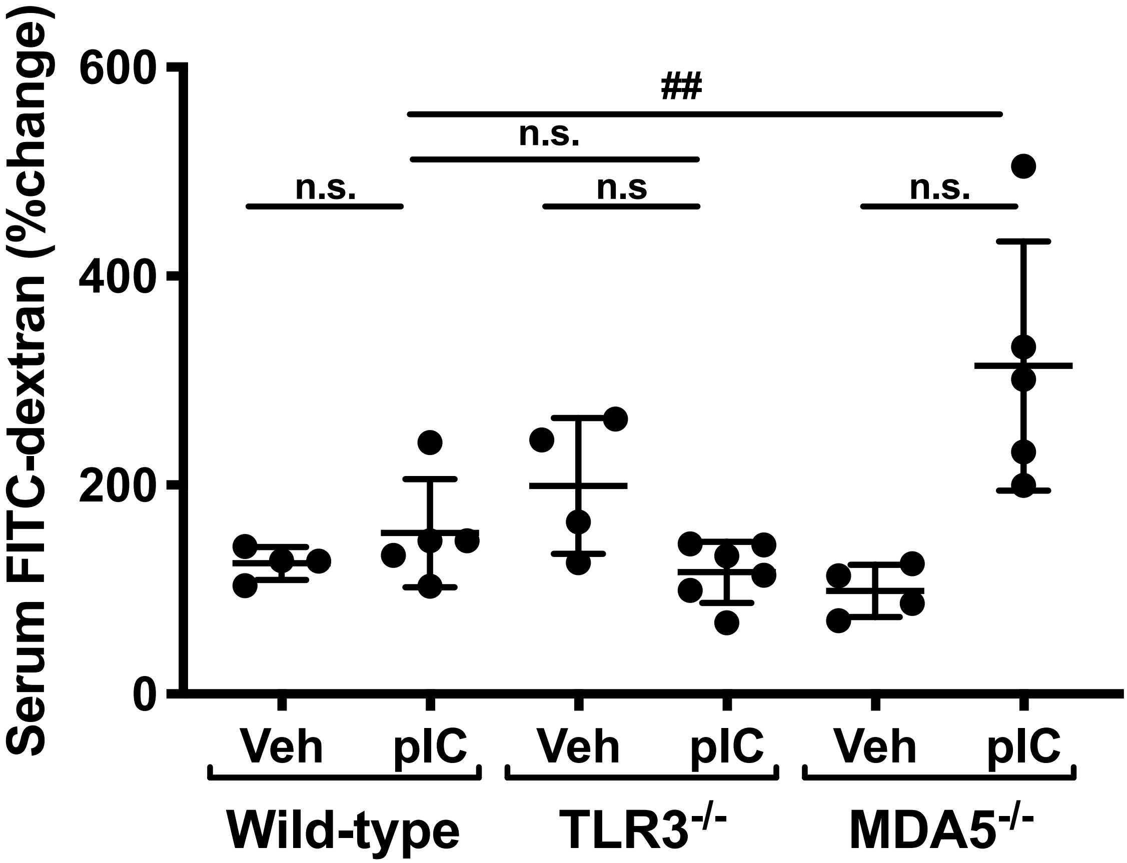

Figure S2 legend. Serum levels of FITC-dextran in wild-type, TLR3-deficient, and MDA5-deficient mice. Mice were challenged with vehicle or inhaled polyI:C (pIC) for three days, and then injected i.p. with 4 kDa FITC-dextran (5 mg) one hour prior to sacrifice. Serum was collected and analyzed for realtive fluorescence as in the Methods section. n.s. = not-significant. ## p<0.01.
